# Customer support for nudge strategies to promote fruit and vegetable intake in a university food service

**DOI:** 10.1186/s12889-022-13054-7

**Published:** 2022-04-10

**Authors:** Sunghwan Yi, Vinay Kanetkar, Paula Brauer

**Affiliations:** 1 Department of Marketing & Consumer Studies, University of Guelph, 50 Stone Rd E, Guelph, ON N1G 2W1 Canada; 2 Department of Family Relations & Applied Nutrition, University of Guelph, 50 Stone Rd. E, Guelph, ON N1G 2W1 Canada

**Keywords:** Nudging, Choice architecture, Healthy eating, Fruits and vegetables, Public support, Perceived intrusiveness, Perceived effectiveness, Food choice environment

## Abstract

**Background:**

Diverse nudges, also known as choice architectural techniques, have been found to increase fruit and vegetable (FV) selection in both lab and field studies. Such strategies are unlikely to be adopted in mass eating settings without clear evidence of customer support; confirmation in specific contexts is needed. Inspired by the Taxonomy of Choice Architecture, we assessed support for eight types of nudging to increase the choice of FV-rich foods in a university food service. We also explored whether and to what extent nudge support was associated with perceived effectiveness and intrusiveness.

**Methods:**

An online survey was conducted with students who used on-campus cafeterias. Multiple recruitment methods were used. Participants were given 20 specific scenarios for increasing FV selection and asked about their personal support for each nudge, as well as perceived intrusiveness and effectiveness. General beliefs about healthy eating and nudging were also measured. Results were assessed by repeated measures ANOVA for the 8 nudge types.

**Results:**

All nudge scenarios achieved overall favourable ratings, with significant differences among different types of nudging by the 298 respondents. Changing range of options (type B3) and changing option-related consequences (type B4) received the highest support, followed by changing option-related effort (type B2) and making information visible (type A2). Translating information (type A1), changing defaults (type B1) and providing reminders or facilitating commitment (type C) were less popular types of nudging. Providing social reference points (type A3) was least supported. Support for nudge types was positively associated with the belief that food services have a role in promoting healthy eating, perceived importance of FV intake, trustworthiness of the choice architect and female gender. Lastly, support for all types of nudges was positively predicted by perceived effectiveness of each nudge and negatively predicted by perceived intrusiveness above and beyond the contribution of general beliefs about healthy eating and nudging.

**Conclusions:**

Findings from the current study indicate significant differences in support for nudge techniques intended to increase FV selection among university cafeteria users. These findings offer practical implications for food service operators as well as public health researchers.

**Supplementary Information:**

The online version contains supplementary material available at 10.1186/s12889-022-13054-7.

## Background

Since the publication of Thaler and Sunstein’s [1] pioneering work on nudging to promote health and wellness choices, evidence of its effectiveness has been accumulating. Nudging refers to a broad set of strategies that alter aspects of the immediate choice environment (i.e., choice architecture), which do not involve substantial economic incentives (e.g., fines or subsidy) or ban alternatives [1]. Foundational to the potential success of such approaches is the extent to which people will support or oppose the use of different types of nudging in various domains. This line of research is of practical importance, in that even if a certain nudge is effective in pilot studies, policy makers or choice architects are likely to avoid putting it into practice if a sizable minority of people oppose it [2].

In food service contexts, operators depend on customer support to maintain sales, and concerns about negative customer reaction were prominent in a key informant study of post-secondary food services managers’ opinions about various nudges to promote fruits and vegetables (FV) [3]. In contexts where customers do not have a choice, such as in schools or worksites, such concerns may be less prominent, but negative customer reaction can still be damaging, in terms of efficiency, reputation and willingness to experiment. Thus, evidence “a priori” on potential customer support is an important element in considering various nudging strategies to promote healthier diets in many food choice settings. Furthermore, this line of research is of potential theoretical importance if certain types of nudges are supported or opposed by significant subgroups in the population that share certain characteristics or psychological processes [4].

It has been common in managing food issues to use traditional policy options, such as regulation on content, outright bans or decreasing availability of unhealthy options, taxation or financial incentives, and informational campaigns. However, public support has been found to differ across policy options. For example, in an early systematic review of public support for government interventions, Diepeveen et al. [5] reported that public acceptance of government interventions intended to change several health-related behaviours was lower for the ones that restricted or eliminated choices, which were generally regarded as intrusive or limiting one’s freedom. In comparison, financial incentives, informational campaigns or warning labels received higher support despite relatively low effectiveness [6]. In a multi-country survey of public support of food policies designed to promote healthy eating, Kwon et al. [7] similarly found that banning, taxing and other restrictions to snack foods received substantially lower approval than subsidies for healthy food choices, provision of calorie information and interventions aimed at children. One of the reasons nudging is a promising policy tool is that it is generally regarded as less intrusive, while producing more cost-effective outcomes than heavy-handed policies or educational campaigns [8].

### Public support for nudging

Empirical findings to date on public support for nudging suggest that although many nudges are supported by most people, some nudges receive substantially less support than others. For example, Hagman et al. [2] asked Swedish and American participants to indicate their support for 10 nudges in different domains, such as placing healthy food items at eye-level for cafeteria users, a comparison of energy consumption between the customers’ household and other households in the neighborhood with a happy or sad face in monthly energy bills, etc. All the nudges received majority support in both countries, except for nudges involving changing defaults, namely automatic registration as organ donors (with the option of opt-out) and automatic registration to pay a climate compensation fee when buying flight tickets (with the option of opt-out). These two default-changing strategies were opposed by about 55% of American participants and by slightly less than 40% of Swedes.

Reisch and Sunstein [9] drew a multinational sample representing six European countries and asked them whether they supported or opposed 15 nudge strategies from various domains. Some nudges were purely informational (e.g., placing warning labels on food with high salt content), whereas others involved changing defaults (e.g., automatic registration for organ donation). Although there were some differences across countries, most nudges received high support except for those involving changing defaults of payment for worthy causes with opt-out provisions (i.e., carbon emission compensation fee for flights or donation to the Red Cross for tax return). Having established such differences exist, several researchers have begun to examine why some nudges receive more support than others.

### Support for different types of nudging

Drawing from the dual process theories’ distinction between mindless versus deliberative mental processing [10–12], Jung and Mellers [13] compared public support for System 1 nudges versus System 2 nudges across domains. System 1 nudging relies on relatively mindless processes (e.g., placing target items closer to customers, changing defaults), whereas System 2 nudging depends on more deliberative and effortful processes (e.g., providing caloric information next to food items or sending timely reminders) [14]. Using two representative samples of Americans, the authors found significantly less support for System 1 nudges involving change of defaults (e.g., people obtaining drivers licenses being automatically enrolled as organ donors unless they chose to opt out) than for System 2 nudges providing information (e.g., requiring credit card companies to provide customers with spending alerts if they are close to a spending limit). Support for System 2 versus 1 nudging was found to be moderated by individual differences in empathy, reactance, etc. However, some System 1 nudges received high support, such as placing salads and lower calorie foods rather than not-so-healthy foods closer to students in school cafeterias.

Within the food choice/eating domain, researchers have recently started to assess support for different types of nudging interventions along with relevant values and attitudes, such as their perceived intrusiveness, fairness and effectiveness and/or trustworthiness of the source of intervention. For example, Petrescu et al. [15] found that support for interventions intended to reduce sugar-sweetened beverages was positively associated with their perceived effectiveness. Furthermore, it was found that public support for nudging strategies (e.g., reducing portion size, changing shape of the drinking container and changing shelf-location for sugar-sweetened beverages) was significantly higher than support for taxation but lower than support for educational campaigns.

In a survey with participants from the US and seven European countries, Evers et al. [16] investigated citizens’ approval of three nudges intended to promote healthy eating: placing healthy foods in more visible places (i.e., at eye level or near the cash register) in grocery stores, making healthier snack options more available/accessible, and placing smaller plates for diners to help them eat smaller portion sizes. Overall, the nudge involving plate size received significantly lower support than nudges intended to increase visibility and accessibility. Furthermore, the plate size nudge was rated significantly more intrusive than the other two nudges, and perceived intrusiveness partially mediated the lower support for the plate size nudge versus the other two nudges. It was also found that support for nudges was higher when the choice architect was considered trustworthy.

### Correlates of support for types of nudging

Two variables were found to be strongly correlated with support for nudge types in recent studies: perceived effectiveness and perceived intrusiveness. Cadario and Chandon [17] conducted an online survey, where 118 American participants were asked to indicate their support for and perceived effectiveness of 7 types of healthy eating nudges [18]: descriptive labelling, evaluative labelling, salience enhancement, healthy eating calls, hedonic enhancements and convenience enhancements. The question on perceived effectiveness was: “Knowing that people are supposed to eat about 2000 cal per day, please estimate the amount of calorie reduction that this policy would lead to”. They were also asked to indicate perceived primary beneficiary of each nudge (i.e., business, consumer health, win–win). Each type of nudge was presented with a brief explanation and a real-life example in healthy eating contexts. The authors found that descriptive labeling and evaluative labeling, which are mainly intended to provide information, received the highest approval as well as the highest *perceived effectiveness* ratings. In contrast, portion size reductions and convenience enhancements, which utilize change of defaults and thus likely involve little deliberation, received substantially lower approval and perceived effectiveness in helping people reduce caloric intake. Cadario and Chandon also found that for all the nudge types, most participants felt that perceived primary beneficiary of the nudge would be customers’ health or win–win. However, a noticeable percentage of customers responded that business would be the primary beneficiary for size enhancements and hedonic enhancements.

Lastly, Djupegot and Hansen [19] asked a representative sample of Norwegian residents to rate their support of 5 placement-changing nudges and 6 information provision strategies intended to increase the choice of healthy foods or to decrease the choice of unhealthy foods in cafeteria settings. Participants were also asked to rate perceived effectiveness and intrusiveness of each strategy. Separate regressions were used to assess the contribution of *perceived effectiveness* vis-à-vis *perceived intrusiveness* to support rating of each nudge. For each nudge, perceived effectiveness was more strongly associated with support ratings than (the lack of) perceived intrusiveness although both were significant predictors of support. One limitation of Djupegot and Hansen’s study was that their selection of nudges was limited to only changing visibility of target food items and using labels for (un-)healthiness. Furthermore, three of the six information-based strategies were about persuading the public about the importance of healthy eating via TV commercials, information campaigns or posters, and therefore, they were not necessarily nudging but persuasion-based communication strategies. Lastly, although one would expect individual differences in personal beliefs, attitudes or values about healthy eating and food services may be relevant to support for nudging, only gender was included in Djupegot and Hansen’s survey. This prevented researchers from assessing the unique contribution of perceived intrusiveness and perceived effectiveness to support of nudging relative to the contribution of individual difference variables.

### Limitations of empirical studies to date

Our review indicates that nudging to promote healthy eating is overall supported by participants in surveys to date. However, some nudges received greater support than others. Unfortunately, no types or classes of nudges consistently received greater support across domains, in part because nudge techniques used in previous surveys were often chosen in ad hoc manners [2, 9, 20]. Even in studies that examined public support for nudges in a single domain (i.e., promotion of healthy eating), a small set of nudge types were employed [16, 19], which limits our understanding of potential variability in public support for different types of nudging. Although Cadario and Chandon’s [18] study is laudable in that it assessed public support for 7 types of food-related nudges, wording of nudge statements focused on caloric reduction and not promotion of a class of foods (e.g. fruits and vegetables), which undoubtedly affected support ratings. Furthermore, although pre-commitment and social referencing have been identified as potent strategies in some typologies of nudging [21, 22], neither has been included in previous studies that assessed support for nudging.

Given the limitations of empirical studies to date, it is necessary to compare public support for a wide range of nudging types within a single domain and a specific context. We chose to survey customers’ perceptions of the use of different types of nudging in fruit and vegetable (FV) promotion in mass-eating settings. A specific focus on increased FV consumption is justified by the large body of epidemiological evidence relating increased consumption to decreased mortality [23, 24]. In adults, most public health agencies worldwide have recommended at least 400 g or five portions of FV per day [25]. The majority do not meet these recommendations, including young adults. Given that university cafeterias serve meals to a large number of students and staff members on a daily basis [26], the implementation of nudging in order to increase their choice of FV in mass eating contexts is deemed a promising application of the concept. Although various types of nudging intended to increase the choice of FV have recently been effective in lab and field studies (for reviews, see [27–29]), effective nudging techniques may not be put into use if food service operators fear that a sizeable number of customers are not in support of them and may negatively react to them [30].

### Taxonomy of nudging techniques

To organize diverse nudging strategies, we compared several recently proposed taxonomies of nudging or techniques of choice architecture, including the TIPPME [31], MINDSPACE [32] and 7 categories of nudging for healthy eating [18]. Since Münscher et al.’s [33] Taxonomy of Choice Architecture (TCA) was deemed to cover more diverse nudge types than the other taxonomies (e.g., translating information, facilitating commitment, changing range of options), we selected it as the guiding framework in preparing the set of nudging strategies for our survey. TCA consists of 3 high-order classes of nudging: (A) the class of techniques that target the presentation of decision-relevant information without altering the choice alternatives themselves (i.e*., decision information*), (B) the class of techniques that alter the structure of choice alternatives and the decision making format (i.e. *decision structure*), and (C) the class of techniques that provide individuals with assistance to help them stick to their intentions to choose “better” alternatives (i.e., *decision assistance*). Furthermore, nine types of specific nudge strategies are specified under the three high-order classes in TCA (see Table 1).

**Table 1:** Nudging types and strategies based on Taxonomy of Choice Architecture (TCA) [33]

Nudging Type	Descriptor from TCA	#	Strategy
A1	Translate information (e.g., simplifying or making information more familiar or attractive)	1	Names for FV-rich menu items (e.g., Grilled green peppers) are replaced with more attractive names, such as “Crunchy Southwestern grilled green peppers”^a^
A2	Make information visible (e.g., providing information at the right moment that may help individuals make informed choice)	2	FV-rich product packaging is now clearly indicated with either “Health check” or “Traffic light” symbols (e.g., green light for healthy option, yellow light for caution; red light for less healthy option)
		3	All food items available in campus food locations have calories on menu boards so that users can take this into account when choosing foods
		4	FV-rich food items are now placed at the beginning or end of menu boards in order to make them more visible
		6	Symbols promoting FV-rich food items (e.g., Health checks, Guiding Stars) are placed on MENU BOARDS
A3	Provide social reference point (e.g., referring to descriptive norms or individuals considered opinion leaders)	5	FV-rich food items are endorsed by famous athletes, chefs, media influencers or local celebrities
B1	Change default (e.g., setting no-action default so that disinterested individuals may opt out and prompting individuals to choose a “better” alternative)	7	FV-rich sides, such as broccoli spears or coleslaw, are now prominently displayed and offered as the “default” or "usual" side to main entrées. A less healthy side, such as French fries, is still available, but you have to ask for it
		8	A newly designed plate is introduced on campus. The plate is divided into three sections, one of which is coloured green and intended to show the recommended amount of vegetables and fruits (i.e., "half your plate"), compared to meat/protein-rich foods and starchy foods
		11	For self-service stations featuring FV-rich foods (e.g., salad bars), big plates are placed in front and small/medium plates are placed in the back to encourage more selection of FV-rich foods
		12	For self-service stations featuring foods generally considered NOT so healthy, small/medium plates are placed in front and larger plates are placed in the back in order to encourage selection of smaller portions of these foods
B2	Change option-related effort (e.g., reducing physical effort necessary to reach or choose a “better” alternative)	9	A station that features FV-rich foods, such as the salad bar, is to be moved to a central location in cafeterias or dining halls
		10	FV-rich foods are moved to the beginning of a buffet or cafeteria line for self-selection
		13	FV-rich food items are displayed in areas that are more noticeable, such as eye-to-waist level of shelves, brightly lit displays, or near checkout lines
B3	Change range of options (e.g., change categories or grouping of target alternatives so that they are perceived as more dominant or attractive)	14	Stir-fry and/or pasta stations now offer a significantly greater range of FV additions and options than before
		15	Some on-the-go sandwiches or wraps (pre-prepared and packaged) now include more vegetables than before
B4	Change option consequences (e.g., connect decision to benefit or offer micro-incentives)	16	Loyalty cards are introduced such that small incentives are provided in return for frequent purchase of FV-rich foods (e.g., one free whole fruit such as an apple or banana, after 5 eligible FV purchases)
C1	Provide reminders (e.g., reminding individuals of their intention at the right moment)	17	Small posters are displayed at food stations in on-campus food locations, promoting more FV purchases
		18	An app is launched on-campus that can be downloaded to cell phones that messages students about the FV-rich food options available 1–2 h in advance of traditional meal times
C2	Facilitate commitment (e.g., supporting commitment to the self-imposed goals)	19	Another app is launched on-campus that can be downloaded to cell phones that will track users’ purchase of FV-rich foods bought on campus and provides feedback on FV intake on campus for that week. (Would be available for interested students only)
		20	An app is launched on-campus that can be downloaded to cell phones that prompts users to set a personal GOAL for FV servings per day (or week), and provides weekly feedback on success based on on-campus purchase record. (Would be available for interested students only)

^a^Although this nudge does not involve logically equivalent reframing (e.g., gain vs. loss framing), it is regarded as reframing in a wide sense in that it is intended to shift the focus of cafeteria users by presenting the description of menu items in a more attractive manner

### Research questions

In this exploratory survey research, the primary aim was to compare support for a wide range of nudge strategies intended to increase the choice of FV-rich food items [33]. Furthermore, we aimed to explore general beliefs about healthy eating and nudging as well as nudge type-specific perceptions that may predict support for different nudge types. Inspired by Evers et al. [16], we explored the possibility that support for certain nudges may be associated with the perceived trustworthiness of the of the food service operator and/or their role in promoting healthy eating (i.e., the belief that food service operators should actively promote healthier food choices or refrain from influencing food choices). Similarly, support for certain nudge types may be more strongly supported by individuals who believe frequent FV intake is important or among those whose daily intake is higher than others. Furthermore, given that perceived effectiveness [16] and perceived intrusiveness [17, 19] of nudges have been strongly associated with support ratings in past studies, we decided to assess the extent to which support for nudge types were associated with perceived effectiveness and intrusiveness *above and beyond* the contribution of general beliefs about healthy eating and nudging. We explored these questions in a survey with users of on-campus food service cafeterias of a Canadian university.

## Methods

### Preparation of nudge statements to be used in the survey

We prepared a list of 20 specific nudge strategies intended to increase the choice of vegetable-rich food items in campus cafeteria settings. Some of the nudge strategies in our list were borrowed from previous publications on the use of nudging to promote FV [18, 27, 28] and from our previous studies about promoting FV in on-campus cafeterias [26, 34]. Other nudge strategies were adapted from previous research that applied nudging to domains other than FV promotion, such as encouraging physical exercise or cutting down on smoking [13, 35–37]. The nudge strategies collected this way were categorized by consensus of the researchers into one of the 9 types of nudges from Münscher et al.’s [33] TCA (See Table 1). For example, moving a station featuring FV-rich foods to a central location, moving FV-rich food items to the beginning of a buffet or cafeteria line, and displaying FV-rich food items in more noticeable areas were categorized into B2 “Changing option-related effort”. As a result, multiple statements were assigned to TCA types of A2, B1, B2, B3, C1 and C2, while there was one nudge strategy statement for each of A1, A3 and B4.

### Participants

Undergraduate students who sometimes or often ate meals at on-campus cafeterias were recruited via non-probability sampling to the online survey (Qualtrics XM Inc. https://www.qualtrics.com/core-xm/survey-software/) in February and March 2020 (i.e., pre-COVID-19 lockdown). Participants were recruited multiple ways: by posting the link to the survey on several websites and University Facebook groups, as well as on course websites for various undergraduate programs offered by the university. Participation in the research was completely voluntary, and no course credit was offered in return for participation. Participant names were entered into a draw for a $100 prize, and the odds of winning were 1/20. Students were eligible to take the survey if they regularly purchased meals from cafeterias or dining halls on campus. If they only purchased beverages or simple snacks (e.g., cookies and muffins) on campus, they were not eligible.

### Survey

The online survey included the following measures as well as demographic questions, such as gender, age, the number of semesters completed, the program of study, the frequency of eating meals or snacks on campus, having meal plans, special diet they practice and food allergies, which were asked at the end. Questions were adapted from previous surveys and reviewed by six undergraduate students who underwent in-person cognitive interviewing via the “think aloud” approach while completing the online survey [38]. The final questionnaire is available from the corresponding author.

#### Support, perceived effectiveness and perceived intrusiveness of 20 nudges

A short description of each of 20 nudges (See Table 1) was provided to participants, one at a time. Participants were asked to indicate the degree of support for each nudge (i.e., “Would you support this change if it is to be introduced at your university food locations?”) on a 4-point scale (1 = ‘disapprove very much’; 2 = ‘somewhat disapprove’; 3 = ‘somewhat approve’; 4 = ‘approve very much’).

Then participants indicated perceived effectiveness of each nudge on their peers’ food choice (i.e., Do you think this change would influence OTHER STUDENTS to choose more FV-rich items if introduced at your university food locations?”) on a 4-point scale (1 = ‘not at all likely’; 2 = ‘somewhat unlikely; 3 = ‘somewhat likely’; 4 = ‘very likely’). Lastly, they were asked to indicate the degree of perceived intrusiveness of each nudge (i.e., “How intrusive would you find this change if introduced at your university food locations?”) on a 4-point scale (1 = ‘not at all intrusive’; 2 = ‘somewhat not intrusive; 3 = ‘somewhat intrusive’; 4 = ‘very intrusive’).

#### Trustworthiness of nudger/choice architect

Participants were asked the extent to which the nudge ideas were designed and implemented out of concern for people’s well-being and health on a 5-point scale (1 = ‘not at all important’; 2 = ‘slightly important’; 3 = ‘moderately important’; 4 = ‘very important’; 5 = ‘extremely important’).

#### Beliefs about food service’s role in promoting healthy eating

Participants were asked whether food services (FS) at their university should actively promote healthier food choices on a 5-point scale (1 = ‘strongly disagree’; 2 = ‘somewhat disagree’; 3 = ‘neither agree nor disagree’; 4 = ‘somewhat agree’; 5 = ‘strongly agree’).

#### Perceived importance of frequent intake of FV

Participants were asked the degree of importance of including a lot of FV in their diet on a 5-point scale (1 = ‘not at all important’; 2 = ‘slightly important’; 3 = ‘moderately important’; 4 = ‘very important’; 5 = ‘extremely important’).

#### Self-report of number of servings of daily FV intake

Participants were asked to indicate the number of servings of total fruit, fruit juice, total vegetables, dark green/orange vegetables and legumes they ate per day over the past week. The questions were adapted to assess daily consumption in the past week, using categories and serving sizes from the 2007 Eating Well with Canada’s Food Guide [39]. One serving was defined as 1 whole fruit or 125 mL (~ 100 g) of fresh or frozen fruits or 100% fruit juice. For total vegetables and dark green/orange vegetables, one serving was defined as 125 mL (~ 100 g) of fresh, frozen or cooked vegetables or 250 mL of raw leafy vegetables. For legumes, one serving was defined as 175 mL of dried beans, lentils, chickpeas. The response options ranged from zero to six servings per day. The index of FV intake was calculated by adding the number of daily servings for total fruit, 100% fruit juice and total vegetables.

### Procedures

When participants logged into the survey, they were asked to read informed consent information on the front page. Once they agreed to participate in the study, they proceeded and filled out the survey at their own pace. On average, it took 13.2 min (SD = 5.5) to complete the survey. Three hundred ninety-five respondents began the survey; about 15% of them dropped out immediately after answering questions on the first online screen; an additional 10% of respondents failed to answer questions about their support of nudge statements. After removal of these participants, the usable data points were *N* = 298 (i.e., 75.4% of those who started the survey). The data collection was approved by the University of Guelph Research Ethics Board.

### Analysis

We conducted statistical analyses using the IBM SPSS Statistics for Windows, Version 28.0. Support ratings for the 20 nudge statements were grouped into eight TCA [33] nudge types, with C1 and C2 types combined. Both C1 and C2 share the same theme of helping individuals stick to their intention. Cronbach alpha was calculated as a measure of internal consistency of support ratings for each nudge type that consisted of more than one nudge strategy statement. The same eight types were used to create indices of perceived effectiveness and perceived intrusiveness of each nudge type. Since each participant rated the whole set of nudge statements, repeated measures ANOVA with the nudge type as one independent variable (IV) was used to compare support ratings, perceived effectiveness and intrusiveness across nudge types.

As a preliminary analysis, we computed Pearson zero-order correlations between personal support ratings of nudge types with a set of major variables, such as their own perceived effectiveness and intrusiveness as well as general beliefs and practices about healthy eating and nudging (i.e., trustworthiness of choice architect, perceived importance of FV intake, the belief that FS should actively promote healthy choices, and self-reported daily number of servings of FV).

To explore how support for nudge types was associated with the four general beliefs and practices abut nudging and healthy eating, we used repeated measures ANOVA, in which support for the 8 nudge types was the repeated measure dependent variable (DV), and effectiveness and intrusiveness were specified as within subject independent variables (IVs) and the general belief variables were specified as between-subject IVs.

Lastly, in order to explore the contribution of perceived intrusiveness and perceived effectiveness of a nudge type to its support while taking into account the associations between nudge support and a set of individual difference variables (i.e., gender, perceived importance of healthy eating, belief that proposed changes are designed out of concern for people’s well-being and health, and belief that FS should actively promote healthier food choices), we ran two-step hierarchal regression analyses with support for each nudge type as the DV, individual difference variables in the first step and perceived intrusiveness and effectiveness in the second step. Running separate regressions for 8 types of nudges fails to take into account intra-individual correlations among ratings of nudge types provided by the same participant. Although multi-level analysis is generally more appropriate to this type of data structure, we did not have any a-priori assumptions about intercepts and slopes of the model to be estimated due to the exploratory nature of the current study.

## Results

### Sample description

Among the 298 participants who completed the survey, 233 (78.2%) were female and 46 (15.4%) were male. Most participants (96.5%) were 19–25 years of age. Most participants (63.8%) identified themselves as Caucasian, followed by East Asian (12.4%), South Asian (8.7%), Arab (5.4%), African/Caribbean (3.7%), Latin American (2.3%), and other (3.7%).

About 19% of participants were first-year students, 31.5% were in their second-year, 21.2% were third-year, 28.3% fourth-year or above. Almost half of participants (47.7%) reported eating one meal or snack on campus (not counting the purchase of only coffee, tea or soft drinks) on an average school day, 18.6% ate two meals/snacks, 12.2% three or more meals/snacks, and 21.5% ate zero meals/snacks. About 37.1% of participants reported having a meal plan in the current semester.

The majority (64.8%) reported not following any special diet, 9.4% were vegetarian, 5.4% were vegan, 3.7% were not eating certain foods due to religious reasons and 1.3% gluten-free. About 10.3% of participants reported one or more types of food intolerance or allergies, such as lactose intolerance, nuts/seeds, fish allergies including food items not tolerable due to religious reasons or personal beliefs.

In terms of their opinion about food on campus, most participants (77.9%) somewhat or strongly agreed that the FS on campus addressed food safety issues adequately. A majority (71.5%) somewhat or strongly agreed that healthy food is readily available on campus. The remainder were neutral or disagreed.

### Descriptive statistics: general beliefs and practices about nudging and healthy eating

#### Trustworthiness of choice architect

More than half of participants (55.2%) strongly agreed that the nudge ideas were designed and implemented out of concern for people’s well-being and health (32.7% somewhat agreed, 12.1% neutral or disagreed).

#### Beliefs about FS’s role in promoting healthy food

About 37.7% strongly agreed and 46.3% somewhat agreed that the FS should actively promote healthier food choices (46.3% somewhat agreed, 12.1% neutral, 4.0% disagreed).

#### Perceived importance of frequent intake of FV

About half of participants (51.2%) reported that it was extremely important to include a lot of FV in diet (35.2% indicated it was very important, 13.5% moderately or less important).

#### Self-reported daily servings of fruits, 100% fruit juice and vegetables

Overall, participants reported an average daily intake of 5.4 servings of fruits, 100% fruit juice and vegetables (95% confidence interval 5.1 to 5.74 servings). The average FV intake reported by our participants was comparable to population average intakes, although methods of diet assessment differed (food frequency vs. 24 h recall) [40].

### Descriptive statistics: support for nudge types, perceived intrusiveness, perceived effectiveness

As five nudge types consisted of more than one items, we calculated Cronbach alpha as a measure of internal reliability. Cronbach’s alpha was 0.76 for changing option-related effort (Type B2), 0.71 for decision assistance nudge type (Type C), 0.69 for changing default (Type B1), 0.65 for making information visible (Type A2) and 0.60 for changing range of options (Type B3). Thus, the internal reliability of the nudge types was acceptable [41].

Support ratings for the 8 types of nudging (DV) were first entered into repeated-measures ANOVA with one IV (i.e., nudge types). The multivariate test indicated that there was a significant effect of nudge type on support ratings (Wilks’ Lambda = 0.321, F (7,271) = 82.00, *p* < 0.001), rejecting the null hypothesis that support ratings for the eight nudge types were the same. For post-hoc comparison of means, Bonferroni correction was used to control for multiple comparisons.

**Fig. 1:**
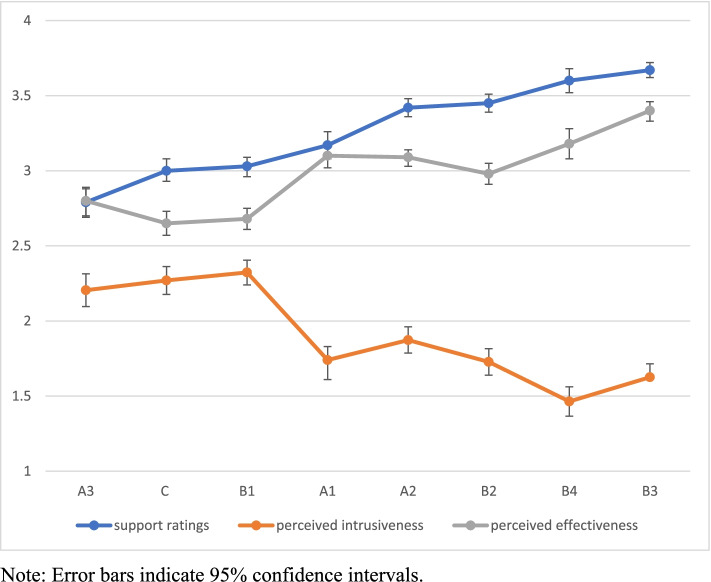
Means of support ratings, perceived intrusiveness and perceived effectiveness for nudge subtypes

Means of support for eight nudge types are listed in Table 2 and plotted in Fig. 1. B3 (changing range of options) and B4 (changing option-related consequences) received the strongest support, followed by A2 (making information more visible) and B2 (reducing effort). In contrast, A3 (social reference group) received the least support. B1 (changing default), C (decision assistance: reminders and facilitating pre-commitment) and A1 (translating information) received significantly lower ratings of support than the other types of nudging, except for A3.

**Table 2:** Means of support ratings, perceived intrusiveness and perceived effectiveness of 8 types of nudging

**Nudge types**		**Nudge order**	**Support Ratings**		**Perceived intrusiveness**		**Perceived effectiveness**	
			Means	S.E	Means	S.E	Means	S.E
A3	provide social reference point	1	2.79^a^	0.05	2.21^a^	0.06	2.80^a^	0.05
C	reminders/commitment	2	3.00^b^	0.04	2.27^a^	0.05	2.65^a^	0.04
B1	change default	3	3.03^b^	0.03	2.32^a^	0.04	2.68^a^	0.03
A1	translate information	4	3.17^b^	0.05	1.74^b^	0.05	3.10^b^	0.04
A2	make information visible	5	3.42^c^	0.03	1.87^b^	0.04	3.09^b^	0.03
B2	change option-related effort	6	3.45^c^	0.03	1.73^b^	0.05	2.98^b^	0.04
B4	change option-related consequences	7	3.60^d^	0.04	1.46^c^	0.05	3.18^b^	0.05
B3	change range of options	8	3.67^d^	0.03	1.63^b^	0.05	3.40^c^	0.03

All were measured with a 4-point scale (4 = approve very much; very intrusive, very effective). Means that bear different alphabet superscripts in the same column were significantly different at *p* < .05 after Bonferroni correction

Likewise, perceived intrusiveness ratings (DV) of the eight types of nudging were entered into repeated-measures ANOVA. There was a significant effect of nudge types on perceived intrusiveness ratings (Wilks’ Lambda = 0.267, F (7,271) = 106.52, *p* < 0.001). The means of perceived effectiveness is listed in Table 2 and plotted in Fig. 1. B1 (changing default), C (reminders and facilitating commitment) and A3 (providing social reference point) were perceived least persuasive although their means were below the mid-point of the scale used (i.e., 2.5). B2 (changing option-related effort), A1 (translating information), B3 (changing range of options) and A2 (making information visible) were perceived as significantly less intrusive than B1, C and A3. B4 (changing option-related consequences) was perceived as significantly less intrusive than any other nudge types.

Lastly, perceived effectiveness ratings of the eight types of nudging were entered into repeated-measures ANOVA. There was a significant effect of nudge types on perceived intrusiveness ratings (Wilks’ Lambda = 0.401, F (7,272) = 58.11, *p* < 0.001). The means of perceived effectiveness is listed in Table 2 and depicted in Fig. 1. B3 (changing range of options) was perceived as the most effective. This was closely followed by a group of B4, A1, A2 and B2 nudge types. By comparison, C, B1 and A3 were perceived as the least effective although their means slightly exceeded the mid-point of the scale used (i.e., 2.5).

### Preliminary analyses: correlations between support for types of nudging and major variables

We computed Pearson correlations between support ratings and perceived intrusiveness/effectiveness ratings for each of the 8 types of nudges. For each nudge type, support rating was positively correlated with perceived effectiveness (0.41 < *r*’s < 0.71, *p*’s < 0.001) and negatively correlated with perceived intrusiveness (-0.52 < *r*’s < -0.29, *p*’s < 0.001). Perceived intrusiveness of nudge types was negatively correlated with perceived effectiveness, albeit weakly (-0.05 < *r*’s < -0.29). However, the correlation was close to zero and not significant for A1 (translating information), B2 (changing option-related effort) and B4 (changing option-related consequences). The table listing correlations is available in Additional File 1 (Supplementary Table 1).

Next, we computed Pearson correlations between support for nudge types and general beliefs about healthy eating and nudging. A table listing these correlations are available in Additional File 1 (Supplementary Table 2). Belief that nudges are designed out of concern for people’s well-being and health was positively correlated with support for all the nudge types (0.14 < *r*’s < 0.35, *p*’s < 0.01). Perceived importance of FV intake was positively correlated with support for all the nudge types (0.16 < *r*’s < 0.42, *p*’s < 0.01) except for A3 (social reference point) (*r* = 0.07, n.s.). Belief that FS should actively promote healthier food choices was positively correlated with support for all the nudge types (0.16 < *r*’s < 0.43, *p*’s < 0.01) except for A1 (translating information) (*r* = 0.07, n.s.). Lastly, self-reported frequency of intake of FV was not significantly correlated with support for all the nudge types except for B1 (*r* = 0.12, *p* = 0.04).

### Support for nudges predicted by beliefs and practices about nudging and healthy eating

Next, we explored the association between support for nudge types and personal beliefs and practices abut nudging and healthy eating by predicting support with the latter variables. Specifically, we used repeated measures ANOVA with four personal variables as between-subject independent variables: the belief that nudging is designed out of concerns for people’s well-being and health (i.e., trustworthiness of choice architect), perceived importance of FV intake, the belief that FS should actively promote healthier food choices, and self-reported daily servings of FV. These variables were entered as continuous variables instead of median-split in order not to reduce the power of analysis. Lastly, gender was added as a between-subject independent variable.

Omnibus multivariate tests indicated that there was a significant main effect of nudge types on perceived intrusiveness ratings (Wilks’ Lambda = 0.82, F (7,261) = 8.16, *p* < 0.001). More importantly, the nudge type by perceived importance of FV intake interaction effect and the interaction effect between nudge type and the belief that FS should promote healthy food choices were significant (Wilks’ Lambda = 0.96, F (7,261) = 2.06, *p* = 0.05; Wilks’ Lambda = 0.91, F (7,261) = 3.59, *p* = 0.001), indicating that support for nudge types were not the same depending on the strength of the two beliefs. The other two 2-way interaction effects were not significant. Lastly, the gender by nudge type interaction effect was also significant (Wilks’ Lambda = 0.94, F (7,261) = 2.38, *p* < 0.02).

Parameter estimates of the four predictors of support for 8 nudge types were then compared (See Table 3). The estimate for the belief in FS role in healthy eating promotion on support ratings was positive for most nudge types, although its size was higher for nudge types that received the lowest average support (i.e., A3, C and B1) than other nudge types. For example, support ratings for A3 (providing social reference point) significantly increased by 0.27 out of the 4-point scale as this belief fell by 1 point. In contrast, the estimate of this belief on support ratings was not significant for B4 (changing option-related consequences) and B3 (changing range of options). Considering that these two nudge types received the highest average support, this suggests that they were highly supported by participants with low or high belief in FS role in healthy eating promotion.

**Table 3:** Coefficient estimates of gender, general beliefs and practices about nudging and healthy eating as predictors of support for 8 nudge types from repeated measures ANOVA

DVs	IVs	*b*	*S. E*	*t*	*p*
A3_support	Intercept	1.41	0.37	3.84	0.00
	Trustworthiness of choice architect	0.09	0.06	1.43	0.15
	Perceived importance of FV intake	-0.03	0.07	-0.38	0.70
	FS should actively promote healthier choices	0.27	0.07	4.07	0.00
	Daily servings of FV	-0.01	0.02	-0.66	0.51
	Gender	0.07	0.14	0.55	0.58
C_support	Intercept	1.12	0.25	4.32	0.00
	Trustworthiness of choice architect	0.16	0.04	3.79	0.00
	Perceived importance of FV intake	0.10	0.05	1.95	0.04
	FS should actively promote healthier choices	0.20	0.05	4.37	0.00
	Daily servings of FV	-0.01	0.01	-0.97	0.32
	Gender	-0.16	0.09	-1.77	0.08
B1_support	Intercept	0.91	0.22	4.06	0.00
	Trustworthiness of choice architect	0.10	0.04	2.48	0.01
	Perceived importance of FV intake	0.22	0.04	5.01	0.00
	FS should actively promote healthier choices	0.20	0.04	5.01	0.00
	Daily servings of FV	-0.01	0.01	-0.57	0.52
	Gender	-0.17	0.08	-2.14	0.03
A1_support	Intercept	2.07	0.35	5.75	0.00
	Trustworthiness of choice architect	0.13	0.06	2.05	0.04
	Perceived importance of FV intake	0.13	0.07	1.87	0.06
	FS should actively promote healthier choices	-0.01	0.06	-0.17	0.87
	Daily servings of FV	0.01	0.02	0.62	0.53
	Gender	-0.07	0.13	-0.53	0.59
A2_support	Intercept	1.72	0.20	8.55	0.00
	Trustworthiness of choice architect	0.14	0.03	4.04	0.00
	Perceived importance of FV intake	0.11	0.04	2.76	0.01
	FS should actively promote healthier choices	0.18	0.04	4.96	0.00
	Daily servings of FV	-0.02	0.01	-1.63	0.08
	Gender	-0.09	0.07	-1.21	0.22
B2_support	Intercept	2.13	0.22	9.82	0.00
	Trustworthiness of choice architect	0.04	0.04	1.20	0.23
	Perceived importance of FV intake	0.19	0.04	4.59	0.00
	FS should actively promote healthier choices	0.11	0.04	2.79	0.01
	Daily servings of FV	-0.02	0.01	-1.57	0.12
	Gender	-0.31	0.08	-4.03	0.00
B4_support	Intercept	2.13	0.30	8.58	0.00
	Trustworthiness of choice architect	0.04	0.05	1.08	0.28
	Perceived importance of FV intake	0.19	0.06	2.72	0.01
	FS should actively promote healthier choices	0.11	0.05	1.13	0.26
	Daily servings of FV	-0.02	0.02	-0.93	0.35
	Gender	-0.28	0.11	-2.61	0.01
B3_support	Intercept	2.82	0.19	15.31	0.00
	Trustworthiness of choice architect	0.06	0.03	1.90	0.06
	Perceived importance of FV intake	0.12	0.04	3.50	0.00
	FS should actively promote healthier choices	0.04	0.03	1.10	0.27
	Daily servings of FV	-0.01	0.01	-0.61	0.54
	Gender	-0.34	0.07	-5.18	0.00

Similarly, the estimate for perceived importance of FV intake on support ratings was positive for most nudge types, indicating that support ratings for them significantly increased (the range was from 0.10 to 0.23) as perceived importance of FV intake rose by 1 point out of the 4-point scale used. However, the estimate of perceived importance of FV intake on support ratings was not significant for A1 (translating information) and A3 (providing social support).

In contrast, the estimate for self-reported number of daily FV servings on support rating was not significant for any nudge type, indicating little variation of the effect of this variable across nudge types. Estimates for the main effect of trustworthiness of choice architect was positive for most nudge types, although its effect on support ratings was relatively small or non-significant.

Lastly, the estimate for the effect of gender on support rating was significant only for B1-B4 nudge types, while not significant for A1-A3 or C nudge type. This indicates that support for B1-B4 nudge types were significantly higher for female participants than males, whereas there was no gender difference for A1-A3 or C nudge types.

Based on median-split, we visualized support ratings of nudge types for participants whose rating of each belief or practice was high versus low. Each figure indicates means of support for 8 nudge types for the high vs. low belief (or practice) group with 95% confidence intervals. They are available in Additional File 2.

### Hierarchical regression analyses: predicting support for different types of nudging

Lastly, we assessed the contribution of perceived intrusiveness and perceived effectiveness of a nudge type to its support while taking account the associations between nudge support and a set of individual difference variables (i.e., gender, perceived importance of healthy eating, belief that proposed changes are designed out of concern for people’s well-being and health, and belief that FS should actively promote healthier food choices). A two-step hierarchical regression was used to examine the contribution of perceived intrusiveness and perceived effectiveness of a certain nudge on its support above and beyond the effect of individual difference variables.

Table 4 lists the results of the two-step hierarchical regression for 8 types of nudges. The explanatory power of Step 1 regression was relatively low, ranging from 0.05 to 0.29. However, the addition of perceived intrusiveness and perceived effectiveness as IVs in step 2 substantially improved adjusted R-squares, which were in the range of 0.41 and 0.68 except for A1 nudge type (0.26).

**Table 4:** Results of two-step hierarchical regressions

DV: Support for nudge type		A3			C			B1			A1	
adjusted R2												
Step 1	0.053			0.185			0.286			0.034		
Step 2	0.425			0.675			0.524			0.255		
Predictors	*b*	*t*	*p*	*b*	*t*	*p*	*b*	*t*	*p*	*b*	*t*	*p*
(Constant)	1.93	5.24	0.00	0.88	3.82	0.00	0.97	3.87	0.00	1.88	4.85	0.00
Gender	-0.05	-0.43	0.67	0.13	2.27	0.02	0.07	1.11	0.27	0.06	0.52	0.60
Perceived importance of FV intake	-0.01	-0.21	0.84	0.07	2.29	0.02	0.12	3.43	0.00	0.04	0.62	0.54
Food services should promote healthier choices	0.08	1.41	0.16	0.08	2.73	0.01	0.11	3.25	0.00	-0.03	-0.47	0.64
FV_servings	-0.01	-0.43	0.67	-0.01	-1.48	0.14	0.00	-0.05	0.96	0.02	0.90	0.37
Designed out of concern for well-being	0.02	0.50	0.62	0.05	1.91	0.05	0.07	2.17	0.03	0.04	0.78	0.43
Intrusiveness of nudge	-0.34	-7.83	0.00	-0.21	-7.11	0.00	-0.22	-6.16	0.00	-0.26	-5.44	0.00
Effectiveness of nudge	0.49	9.71	0.00	0.59	16.24	0.00	0.43	9.55	0.00	0.42	6.96	0.00
DV: Support for nudge type		A2			B2			B4			B3	
adjusted R2												
Step 1	0.233			0.199			0.071			0.176		
Step 2	0.496			0.448			0.410			0.486		
predictors	*b*	*t*	*p*	*b*	*t*	*p*	*b*	*t*	*p*	*b*	*t*	*p*
(Constant)	1.60	6.93	0.00	1.44	6.30	0.00	1.85	6.03	0.00	1.99	10.48	0.00
Gender	0.06	1.03	0.31	0.20	3.02	0.00	0.08	0.93	0.35	0.16	3.00	0.00
Perceived importance of FV intake	0.07	2.23	0.03	0.11	3.09	0.00	0.09	2.07	0.04	0.07	2.46	0.01
Food services should promote healthier choices	0.10	3.19	0.00	0.04	1.19	0.24	0.11	2.52	0.01	0.02	0.81	0.42
FV_servings	-0.02	-1.76	0.08	-0.01	-1.04	0.30	-0.01	-1.01	0.31	-0.01	-1.61	0.11
Designed out of concern for well-being	0.06	1.97	0.05	0.02	0.51	0.61	-0.02	-0.40	0.69	0.01	0.57	0.57
Intrusiveness of nudge	-0.23	-7.41	0.00	-0.15	-4.49	0.00	-0.25	-6.29	0.00	-0.15	-5.57	0.00
Effectiveness of nudge	0.41	8.54	0.00	0.42	9.95	0.00	0.39	10.63	0.00	0.37	10.15	0.00

The findings from Step 1 regression are not reported here since they are very similar to the findings from repeated measures ANOVA reported earlier. The estimates of regression coefficients for predictors in Step 2 regression are shown in Table 4. Overall, # of servings of FV intake was not a significant predictor for any nudge. Gender was significant only for B2, B3 and C type of nudge, which were more supported by females than males. Trustworthiness of choice architect (i.e., designed out of concern for well-being) was a positive predictor of support for A2, B1 and C. Belief that FS should promote healthier food choices was a positive predictor of support for A2, B1, B4 and C. Perceived importance of eating FV was a positive predictor for all types of nudges except A1 and A3.

More importantly, perceived effectiveness and perceived intrusiveness of each nudge were significant predictors of support for all types of nudging above and beyond individual difference variables. Specifically, perceived intrusiveness was a negative predictor of support of all types of nudging (-0.34 < b’s < -0.15, *p*’s < 0.001), whereas perceived effectiveness was a positive predictor (0.37 < b’s < 0.59), *p*’s < 0.001. However, the effect of perceived effectiveness on support of each nudge was substantially stronger than the effect of perceived intrusiveness. For example, with regard to nudge type C, the regression coefficient for perceived effectiveness was quite substantial (b = 0.59, t = 16.24, *p* < 0.001), whereas the coefficient for perceived intrusiveness was small by comparison (b = -0.21, t = -7.11, *p* < 0.001). In contrast, the gap of the size of the two coefficients was smaller for nudge type A3 (b = 0.49, t = 9.71, *p* < 0.001 for perceived effectiveness and b = -0.34, t = -7.83, *p* < 0.001 for perceived intrusiveness). The size of the regression coefficient for perceived effectiveness was especially higher for C and A3 than the other nudge types.

## Discussion

Inspired by the Taxonomy of Choice Architecture [33], we surveyed university students to compare support for a wide range of types of nudges intended to increase the choice of FV-rich food in cafeteria settings. We also explored the degree to which support for nudge types may be associated with their personal beliefs and practices about healthy eating and nudging, such as trustworthiness of motive for implementing nudging, the perceived role of food service operators in promoting healthy eating, the perceived importance of FV intake and their daily intake of FV. Finally, we examined the extent to which support for nudge types was associated with nudge type-specific beliefs, namely, perceived effectiveness and intrusiveness, above and beyond the contribution of personal beliefs and practices about healthy eating and nudging. Although our study was exploratory, our findings provide an important addition to the literature on public support of nudge strategies intended to promote healthy eating.

We found that although no nudge strategies included in our survey were on average opposed (i.e., lower than the mid-point of the 4-point scale), there were significant differences in support for different types of nudging intended to increase FV choice. Changing the range of options (type B3) and changing option-related consequences (type B4) received the highest support, followed by changing option-related effort (type B2) and making information visible (type A2). Translating information (type A1), changing defaults (type B1) and providing reminders or facilitating commitment (type C) were less popular types of nudging. Providing social reference points (type A3) was least supported.

Nudge types receiving high support among our participants generally involved better deals for cafeteria users than currently available, such as healthier food options added to the current offerings (i.e., type B3) and receiving incentives for repeated purchasing eligible FV-items (i.e., type B4). The two nudge types were perceived to be less intrusive and more effective than the others. However, these types of nudging are likely to involve considerable planning and possibly cost from the perspective of FS. Furthermore, since perceived effectiveness and support for nudge ideas does not necessarily guarantee their actual effectiveness in increasing the choice of target behaviour [17], pilot studies will be necessary to evaluate these strategies.

Furthermore, nudge types that rely on increasing salience in the form of reducing effort for choosing healthy food items per se (i.e., type B2; e.g., placing FV-rich items in easy-to-spot or otherwise convenient locations) or making *information* about FV items more visible (i.e., type A2; e.g., traffic-light nutritional labeling) were also popular among our participants. This appears to reflect the perception that these nudges are a relatively moderate modification of the immediate choice environment and therefore not very intrusive upon one’s freedom to choose. For example, from the perspective of cafeteria users, even if healthy food items are placed in a more central or convenient location than before, non-target food items will still be available for selection as before. Similarly, provision of labels denoting healthiness of food items or placing healthy items on the top or bottom of menu boards does not seem to be perceived as making the choice of non-target food items substantially more difficult than before. Considering that these two types of nudging were also viewed as quite effective, they are promising candidates for any food service operator that is willing to implement nudging.

In contrast, the nudge type that changes defaults (i.e., type B1) was not as supported as nudging strategies that reduce effort in choosing healthy items (i.e., type B2). Although the distinction between the two is not recognized by some nudge researchers (e.g., Cadario & Chandon’s [18] seven categories of healthy eating nudges; Holland et al.’s [31] TIPPME typology), changing defaults typically requires users to ask for or go to another location for non-target items that are not displayed in front of them. In contrast, the selection of non-target items appears to be perceived as relatively easy for nudges intended to reduce the effort in selecting target items by varying relative position, without changing defaults. This reasoning is supported by our finding that changing defaults was perceived as one of the most intrusive types of nudging as well as one of the least effective types of nudging by our participants. Changing defaults is one of the best known examples of nudging, especially after the publication of research that compared opt-in versus opt-out systems for registration as organ donors across European countries [42]. However, consistent with our own findings, intervention studies that have employed this nudge type have not always produced favourable behavioural change and has sometimes led to strong reactance among a sizable number of people, who have perceived this nudge type as limiting their freedom of choice [9, 13, 30, 43]. Therefore, nudging that involves changing defaults would need to be carefully designed such that users can easily exercise their freedom to opt out and choose non-target items. In a recent U.S. study [44], when apple slices were offered as the default snack, 87% of 6 to 8 year old children opted out and asked for French fries instead. This finding suggests that when cafeteria users have a strong preference for certain unhealthy items, changing defaults is not likely to produce favourable behavioural change. Changing defaults is likely to be effective when healthy food items to be used as new defaults are equivalent to or superior to non-target items in sensory properties. This poses a unique challenge for food service operators since it requires recipe development for healthy food items that are also hedonically appealing.

Translating information (i.e., type A1) was also one of the less supported nudge types by our participants. This was surprising to us since using attractive names for FV-rich items have been tried previously in the context of primary school lunchrooms with moderate success [45, 46]. This strategy was perceived as not so effective and quite intrusive by our sample of university students. It is possible that this nudge strategy may only work up to early adolescence, whereas it may be perceived as manipulative by adults. Alternatively, although this type of nudge was not popular when described to survey participants, using attractive names for FV-rich items may effectively nudge unsuspecting adult users when implemented in cafeterias. An empirical field or lab study is necessary to examine perceived effectiveness vis-à-vis actual effectiveness of this nudge type. Furthermore, it will be necessary to verify this finding by examining public support for multiple nudge ideas that exemplify more diverse aspects of nudge type A1 (i.e., reframing and simplifying information). For example, refocusing long-term benefits of the choice of FV-rich items to short-term ones or presenting calorie information about certain items in ways that are easy to relate to, such as physical activity equivalent labeling (see [47] for reviews of studies that examined effectiveness of such labeling).

Providing reminders or facilitating commitment (i.e., type C) was also not highly supported by our participants. Since nudge tactics we came up with for type C were adapted from those that had been successfully used to facilitate other behaviours [36, 48], this finding was surprising to us. However, the use of pre-commitment strategies and offering timely reminders in empirical studies has been limited to other behaviours, such as quitting smoking [49] and increasing money in savings [50]. In fact, they have rarely been applied to the context of healthy eating except for a few studies, in which a pre-commitment device was typically combined with financial incentives for grocery shoppers who are willing to participate [51]. Although school children or hospital workers were asked to pre-select entrées for lunch early in the day in a few recent studies [52, 53], participants were free to select from any meal options available for a day and their commitment was very short-term (i.e., daily). Individuals who voluntarily enroll themselves in commitment devices are likely to be aware that their intentions to engage in a target behaviour are frequently foiled by the lack of self-control. Therefore, it is possible that pre-commitment strategies may only work for people who are strongly motivated to achieve a specific behavioural goal (e.g., eating five servings of fruits and vegetables per day). Although healthy eating is increasingly considered important among young adults, many still tend to distance themselves from too much interest in healthy eating and reveal care-free attitudes and behaviour toward diet [54, 55]. Thus, receiving timely reminders about healthy food options or committing to FV-rich meals is not likely to be welcome by most cafeteria users except for a small number of individuals with strong diet-related concerns.

Lastly, providing a social reference point (i.e., type A3) was the least supported nudge type. It was perceived as one of the least effective and most intrusive nudges. Given previous empirical findings attesting to the potency of utilizing social norms in other fields [56], this finding was also surprising. It is possible that our participants failed to appreciate the power of social referencing just by reading the short sentence we provided. Alternatively, convinced that their behaviour solely reflects their free will, people may refuse to acknowledge that their own choices may be influenced by opinion leaders’ endorsement although acknowledging that others may be influenced. Relatedly, social psychologists have consistently shown that people have limited insight as to mental processes that produce their thoughts or choices [57]. Alternatively, sources of endorsement for FV-rich items we specified (i.e., famous athletes, chefs, media influencers or local celebrities) may not have been considered appropriate by our participants. Since we only had one item for this nudge type, it is hard to verify whether our wording of the item caused this response.

Although some previous researchers have argued that System 2 nudging was more supported than System 1 nudging by the public [20], our findings indicate that this appears to be an over-simplification. We believe that these previous findings were either due to using only default-changing tactics as examples of System 1 nudge [9] or failing to distinguish nudges that involve changing defaults and those that simply change salience of target items [13]. Owing to the systematic typology of subtypes of altering decision structure (i.e., B1-B4), which reflect distinct facets of relatively mindless influence, we were able to find that nudges that change defaults are significantly less supported than those that change option-related effort via salience. Furthermore, although nudge types under the category of decision information in TCA (i.e., A1-A3) are considered System 2 nudging, providing social nudging (i.e., A3) was significantly less supported than A1 and A2. Although System 1 vs. 2 nudging appears to be an intuitively appealing umbrella category, public support and perception of subtypes of nudging under this distinction should not be assumed to be similar.

Our findings were interesting in that there were significant gender differences in support for certain types of nudging. Although gender differences have been reported in recent studies on support for nudges [9, 58], consistent findings have been that females give greater support than males across nudge statements. These findings were typically interpreted as females having greater empathetic concerns than males [58]. Therefore, our finding that females indicated significantly greater support for B1-B4 nudge types than males, but not for A1-A3 or C types offers a challenge to the findings so far. Considering that B1-B4 are collectively about the alteration of decision structure intended to promote behaviour change, which are perceived to be quite intrusive, lower support by males is understandable, given evidence for their reduced interest in healthy eating relative to females [59]. In contrast, the lack of gender differences for nudge types involving alteration of information structure (i.e., A1-A3 subtypes) suggests that they are perceived to respect the sense of freedom of choice and are thus generally acceptable. On the other hand, it appears that the lack of gender difference for support for C nudge type involving reminders and commitment facilitation suggests that it was equally disliked by males and females.

Our findings complement Cadario and Chandon’s [17] findings in three important ways. First, the questions on perceived effectiveness focused on different aspects of the broad concept of healthy eating. Cadario and Chnadon asked about estimated calorie reduction while we asked about increasing FV-rich food items offered. All their nudge statements were worded in such a way that the purpose of each nudge type was to increase the selection of lower calorie foods and to decrease the selection of higher calorie foods (e.g., French fries). Thus, participants’ ratings of support were for reduced calorie intake. Secondly, their sample was drawn from American adults involved in the Mechanical Turk platform, while our subjects were students. Reduction in caloric intake is a major preoccupation among adults in the US, where prevalence of dieting among adults was 49% in the National Health and Nutrition Examination Survey, 2013–2016, and lower at 38% among adolescents [60, 61]. Comparable data for Canada are not available. Finally, the distinction between changing option-related effort and changing defaults was not made in Cadario and Chandon’s [18] nudge categories. For example, their category of convenience enhancements includes not only changing defaults (i.e., B1) but also offering pre-sliced or pre-portioned foods for convenience, which would be categorized as changing option-related effort (i.e., B2). Similarly, their category of visibility enhancements includes tactics intended to increase the visibility of information for target food (i.e., A2) as well as those designed to increase the salience of target food and thus reduce option-related effort (i.e., B2). The more fine-grained nudge types from TCA [33] allowed us to reveal that the nudge type of changing option-related effort was perceived as less intrusive and received higher support than the nudge type involving change of defaults. Therefore, we believe that our findings provide more direct evidence for differential support for diverse nudge types, but only related to promotion of FV.

Furthermore, this work identified that support for some nudge types was significantly associated with several personal beliefs and practices about healthy eating although specific patterns were not homogenous across nudge types. Support for three nudge types, such as providing reminders and facilitating commitment (Type C), changing defaults (Type B1) and making information visible (Type A2), was consistently associated with the three major beliefs included in our survey: trustworthiness of choice architect (i.e., the belief that proposed changes were designed out of concern for well-being and health), the belief that FS should actively promote healthier choices, and the perceived importance of FV intake. Given that support for the three nudge types were on average low to moderate, this may indicate that support for them was more polarized than other nudges. In our view, these three nudge types may be quite controversial for those who have weaker beliefs about healthy eating generally, or FV specifically, or who are critical of food services’ use of nudging in cafeterias. Further work is needed to explore these possibilities.

One unexpected finding was that self-reported daily servings of FV was not significantly associated with support for any nudge subtype. While it is reasonable to expect that support for nudging intended to increase FV intake would be higher for people with high daily servings of FV intake, it is possible that they may believe that nudging is not necessary for them. Alternatively, it is possible that subjective estimation of FV intake on an average day may not be accurate, especially on a self-paced online survey of university students with minimal diet-related concerns.

Finally, as expected, perceived effectiveness and perceived intrusiveness of each nudge type were significant predictors of its support even when the association of nudge support and individual difference variables (i.e., gender and belief variables) were accounted for. Across nudge types, support for nudging significantly rose if it was perceived as more effective and as less intrusive. Furthermore, consistent with Djupeot and Hansens’s [19] recent findings, perceived effectiveness was a stronger predictor than perceived intrusiveness for all types of nudges. Although it is tempting to assume that nudges that are regarded as intrusive would be perceived as not effective in helping to increase the choice of FV, the correlation between the two variables was relatively low although its valence was negative. The correlation was not even significant for translating information (A1), changing option-related effort (B2), and changing option-related consequences (B4). This suggests that perceived effectiveness and perceived intrusiveness have largely separate effects on nudge support.

While acknowledging that the findings on the relationship between nudge support and perception are largely consistent with recent studies [16, 17, 19], we believe our findings provide more definitive evidence for the relationship between support for nudge types and public perceptions, in part because we used a wider range of nudge types as proposed by Münscher et al.’s [33] TCA. However, it is important to recognize that perceived effectiveness of a nudge type does not necessarily predict its actual effectiveness. Furthermore, we have to date limited understanding of how perceived effectiveness of nudges is shaped. Although it is possible that perceived effectiveness of a nudge may be determined by its compatibility with lay beliefs on the relative influence of internal versus external factors on decision making [62, 63], very little conceptual or empirical research is available to date.

Coupled with the Cadario and Chandon’s [17] survey findings that nudge types that had large effect sizes on caloric reduction in their previous meta-analysis [18] (e.g., convenience enhancements and size enhancements) were perceived as less effective than those that had smaller effect sizes (e.g., descriptive nutritional labelling, evaluative nutritional labelling, visibility enhancements), our finding that perceived effectiveness of a FV nudge was a strong predictor of its support presents a unique challenge for choice architects (i.e., the management of a food service operator). Although implementing nudge types that are perceived to be effective by the public (e.g., changing option-related effort or making information visible) may be considered appropriate, this is likely to lead to a relatively small effect on the choice of FV items or reduction in caloric intake. Choice architects must also consider that some nudges (e.g., changing defaults or providing reminders and commitment) are also perceived as quite intrusive [64]. More work on perceived vs actual effectiveness and intrusiveness in different food choice/ healthy eating contexts is needed.

### Limitations and future research directions

Our research is not without limitations. We acknowledge common method bias given that all variables were measured in one single survey one after another. Furthermore, since perceived effectiveness and perceived intrusiveness for a nudge were measured immediately after support for the nudge, reported associations among the three variables are likely to have been somewhat inflated. Although we initially considered asking participants to rate support for each nudge first, then to rate perceived effectiveness and perceived intrusiveness afterwards, this would have required greater time to complete the survey. Future studies that address this limitation are necessary to verify our findings.

Although multiple items for a variable are desired to account for measurement error, we were not able to do so for all the variables. We had only one question item for three nudge types (i.e., A1, A3 and B4). Our findings for the three nudge types should be considered preliminary since they may reflect distortion due to our choice of wording or aspects of a nudge subtype that was not adequately captured with our one item. For example, although A3 (providing social reference point) includes not only referring to opinion leaders but also referring to descriptive norms, we did not have a question item for the latter. Similarly, we devised and used only one item to measure trustworthiness of choice architect, perceived importance of FV intake, and the belief that FS should actively promote healthier choices. Our findings related to these variables await replication.

Due to the preliminary nature of the current study, we used separate hierarchical regression to analyze the association of perceived intrusiveness [effectiveness] and support for nudges above and beyond the contribution of individual difference variables. As we acknowledged earlier, the use of multi-level modeling is more appropriate to account for intrapersonal correlations among support ratings for different nudge types. We hope that our findings will be able to inform future researchers to choose appropriate assumptions for intercepts and slopes for multi-level modelling in this line of research.

Lastly, our sample consisted of students attending a single Canadian university with an independent, award winning food service operator. Furthermore, our sample was not a representative sample of the student population attending this university. Therefore, our findings may not necessarily be generalized to other academic institutions with for-profit contract service operators. More diverse sampling is necessary to determine if and what FV nudges are most acceptable to the general public.

### Practical implications

Foodservices for worksites and post-secondary academic institutions often have a dual mandate to generate profits while promoting users’ health. Thus, they may be potentially interested in adopting nudges intended to increase healthy eating. Our findings offer several important implications for the promotion of FV-rich food in the context of mass eating venues. Given our findings regarding superior support for nudges that make information visible, reduce effort related to target items, enlarge the range of options, or offer favourable consequences of choosing target items, cafeteria operators and nutrition professionals are advised to try out favoured approaches in FV promotion.

Furthermore, for nudging to be accepted among users, it is imperative that food service operators build a trust relationship with cafeteria users so that the users believe that any nudge interventions are designed and implemented out of concern for their health and well-being [65]. Relatedly, the importance of trust in marketing mix and choice architecture interventions intended to increase the choice of healthy foods in the context of grocery stores has repeatedly been reported [66]. If users perceive food service operators as placing their own profit ahead of customers’ health and well-being, nudges intended to increase the choice of healthy food items may be misinterpreted as an attempt to promote higher margin items or to exploit customers. This is consistent with a recent framework on implicit social interaction between choice architect and nudgee [67], which emphasized that for nudging to succeed, the choice architect needs to be perceived as benevolent as well as competent in the eyes of nudgees.

We advise that choice architects work closely with representatives of food service users if they wish to test nudge strategies that are known to be perceived as intrusive (e.g., changing defaults). It is recommended choice architects communicate to users the purpose of implementing the nudge strategy as well as evidence of its effectiveness from a previous lab or field study. This could be communicated via websites and posters around the cafeteria (e.g., “Did you notice that veggie-rich items are now offered as default sides? This is one of our new attempts to help you eat more fruits and vegetables…”). A few recent studies have reported that disclosing the use of some nudges to users did not reduce their effectiveness [52–54]. Furthermore, we advise choice architects to share results of a nudge trial with representatives at the end of the trial period so that effectiveness of nudge strategies can be communicated to cafeteria users. Sunstein [7] reported that nudge tactics that were initially perceived as less effective and intrusive received a higher support rating when participants were informed of the evidence for effectiveness.

Lastly, it is important for practitioners to recognize that successful implementation of nudges is currently as much art as it is science. Slight variation in wording or food display may lead to success or complaints about freedom of choice. Nudges that have produced sizable effects in other locations may need to be adapted to the local context as well as prior experiences and expectations of local cafeteria users.

## Conclusion

In sum, although no nudges intended to increase FV-rich items were opposed by our participants, we found that some nudges were significantly more supported than others. Overall, nudges that made information about target items visible, reduced effort, enlarged the range of options, or offered favourable consequences of choosing target items received high support by our participants. In contrast, nudges that changed default, provided timely reminders and facilitated commitment, and translated information via sensory attributes and opinion leaders were not as highly rated. Furthermore, support for certain nudges was significantly higher among participants who already believed that proposed changes were designed out of concern for well-being and health, or that food services should be actively promoting healthier choices or that frequent FV intake was important for them. Lastly, we found that support for all types of nudges was positively associated with perceived effectiveness and negatively associated with perceived intrusiveness above and beyond personal beliefs about nudging and healthy eating.

## Supplementary Information

**Additional file 1:** **Supplemental Table 1.** Correlations between support for nudge types, perceived effectiveness and perceived intrusiveness. **Supplemental Table 2.** Correlations between support for nudge types and general beliefs about healthy eating and nudging.

**Additional file 2.**

## Data Availability

The dataset generated during and/or analysed during the current study is not publicly available due to the clause as to the restriction of sharing the raw data, but it will be available from the corresponding author on reasonable request.
