# Supplementary material for: Customer support for nudge strategies to promote fruit and vegetable intake in a university food service
Source: BMC Public Health. 2022 Apr 10;22:706. doi: 10.1186/s12889-022-13054-7 (PMC8994925; doi:10.1186/s12889-022-13054-7)
Supplement: Supplementary file 1 — Additional file 1: Supplemental Table 1. Correlations between support for nudge types, perceived effectiveness and perceived intrusiveness. Supplemental Table 2. Correlations between support for nudge types and general beliefs about healthy eating and nudging. [file 12889_2022_13054_MOESM1_ESM.docx]

**Additional File 1.**

Supplemental Table 1. Correlations between support for nudge types, perceived effectiveness and perceived intrusiveness.

|  |  | **social reference** | **reminder and pre-commmit-ment** | **changing defaults** | **translate info** | **info more visible** | **changing option-related effort** | **changing option conseq.** | **changing range or composition** |
| --- | --- | --- | --- | --- | --- | --- | --- | --- | --- |
|  |  | A3 | C | B1 | A1 | A2 | B2 | B4 | B3 |
| Support * Perceived intrusiveness | *r* | -0.46 | -0.52 | -0.4 | -0.33 | -0.47 | -0.29 | -0.37 | -0.42 |
|  | *p* | 0.00 | 0.00 | 0.00 | 0.00 | 0.00 | 0.00 | 0.00 | 0.00 |
| Support* Perceived effectiveness | *r* | 0.52 | 0.74 | 0.59 | 0.41 | 0.51 | 0.59 | 0.52 | 0.63 |
|  | *p* | 0.00 | 0.00 | 0.00 | 0.00 | 0.00 | 0.00 | 0.00 | 0.00 |
| intrusiveness* effectiveness | *r* | -0.15 | -0.31 | -0.14 | -0.05 | -0.16 | -0.08 | -0.06 | -0.29 |
|  | *p* | 0.01 | 0.00 | 0.02 | 0.43 | 0.01 | 0.17 | 0.31 | 0.00 |

Supplemental Table 2. Correlations between support for nudge types and general beliefs about healthy eating and nudging.

|  |  | **social reference point** | **reminders and pre-commmit-ment** | **changing defaults** | **translate info** | **info more visible** | **changing effort** | **changing option conseq.** | **changing range or composition** |  |
| --- | --- | --- | --- | --- | --- | --- | --- | --- | --- | --- |
|  |  | A3 | C | B1 | A1 | A2 | B2 | B4 | B3 |  |
| Trustworthiness of choice architect | *r* | .14^*^ | .32^**^ | .30^**^ | .17^**^ | .35^**^ | .21^**^ | .15^*^ | .22^**^ |  |
|  |  |  |  |  |  |  |  |  |  |  |
|  | *p* | 0.02 | 0.00 | 0.00 | 0.00 | 0.00 | 0.00 | 0.01 | 0.00 |  |
| Perceived importance of FV intake | *r* | 0.07 | .26^**^ | .42^**^ | .16^**^ | .29^**^ | .34^**^ | .21^**^ | .27^**^ |  |
|  | *p* | 0.24 | 0.00 | 0.00 | 0.01 | 0.00 | 0.00 | 0.00 | 0.00 |  |
| Belief that FS should actively promote healthier chocies | *r* | .25^**^ | .36^**^ | .43^**^ | 0.07 | .40^**^ | .28^**^ | .16^**^ | .19^**^ |  |
|  | *p* | 0.00 | 0.00 | 0.00 | 0.27 | 0.00 | 0.00 | 0.01 | 0.00 |  |
| Reported daily services of FV | *r* | -0.02 | 0.08 | .12^*^ | 0.08 | 0 | 0.03 | 0.02 | 0.04 |  |
|  | *p* | 0.79 | 0.20 | 0.04 | 0.20 | 0.98 | 0.60 | 0.77 | 0.47 |  |
