# Supplementary material for: Customer support for nudge strategies to promote fruit and vegetable intake in a university food service
Source: BMC Public Health. 2022 Apr 10;22:706. doi: 10.1186/s12889-022-13054-7 (PMC8994925; doi:10.1186/s12889-022-13054-7)
Supplement: Supplementary file 2 — Additional file 2. [file 12889_2022_13054_MOESM2_ESM.docx]

**Additional File 2.**

Means of support ratings for nudging types for people with high versus low belief that food services (FS) should promote healthy food choices


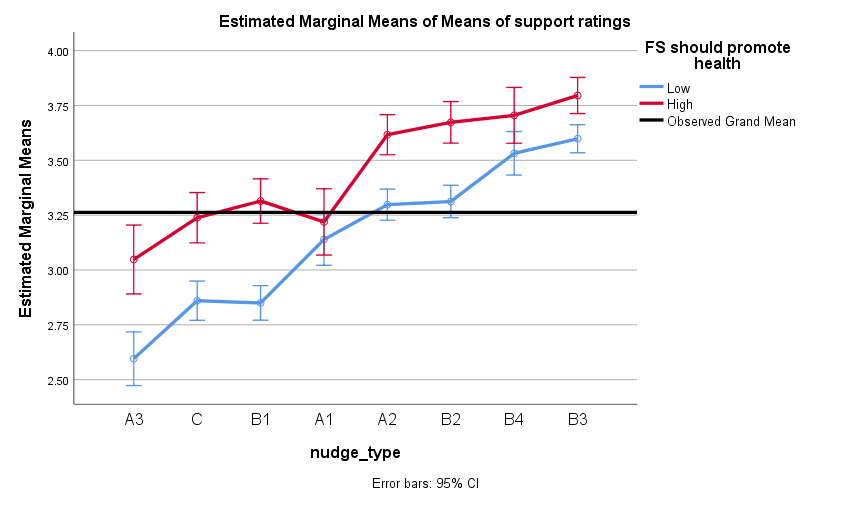


Support ratings of nudging types of A2, B1, B2, B3, C and A3 were significantly higher for people with strong belief that FS should promote healthy food choice than those with weak belief, except for A1 (translating information) and B4 (changing consequences of options).

Means of support ratings for nudging types for people with high versus low perceived important of fruit and vegetable (FV)


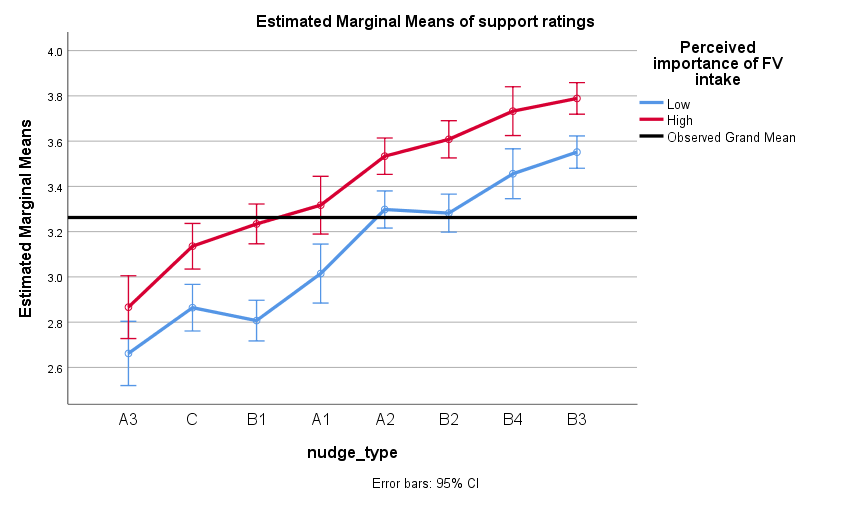


Support ratings for all the types of nudges were significantly higher among those with high versus low perceived importance of FV intake.

Means of support ratings for nudging types for people with strong versus weak belief that nudging is implemented out of concern for users’ well-being.


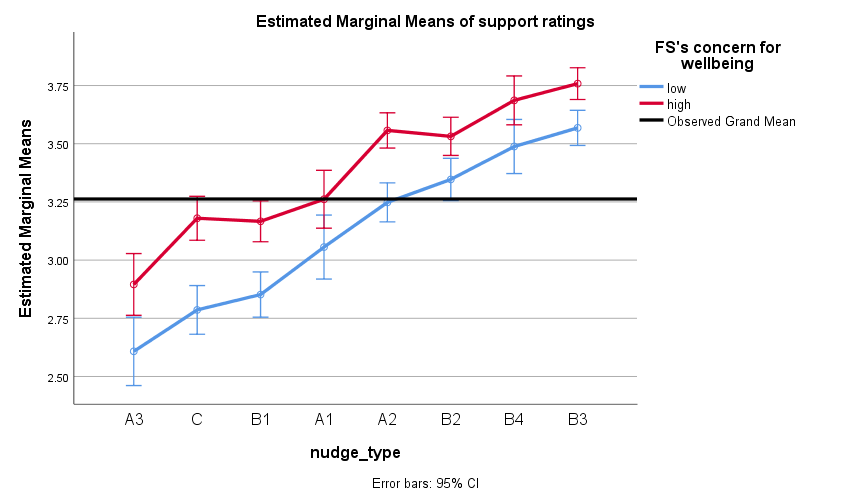


All the types of nudges were significantly higher among those with high belief in nudging implemented to increase users’ well-being versus those with low belief, except for translating information (A1) and changing option-related consequences.

Means of support ratings for nudging types for male versus female


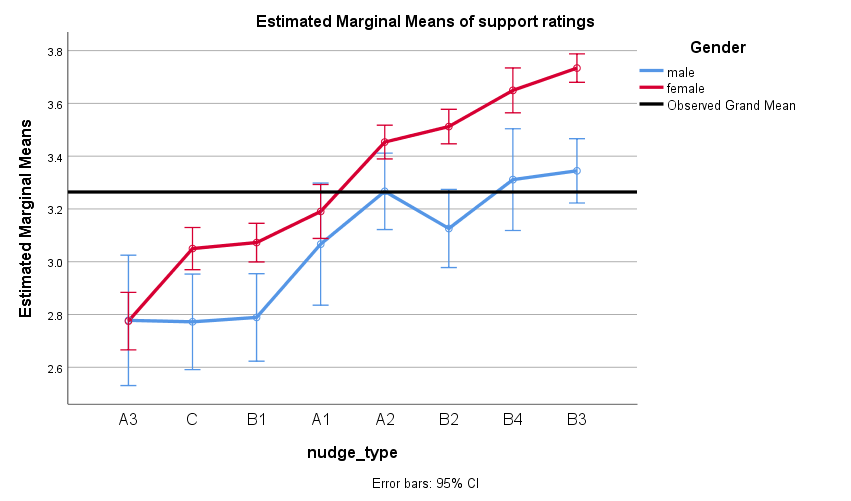


All nudging types received significantly greater support by female participants than males except for A3, A1 and A2 types.
